# Selective serotonin reuptake inhibitors and violent crime: do SSRI’s kill or cure?

**DOI:** 10.1192/j.eurpsy.2022.892

**Published:** 2022-09-01

**Authors:** S. Sneep, S. Petrykiv

**Affiliations:** 1 GGZ Westelijk Noord Brabant, Psychiatry, Halsteren, Netherlands; 2 GGZWNB, Psychiatry, Halsteren, Netherlands

**Keywords:** SSRI, violent behaviour

## Abstract

**Introduction:**

SSRI’s are consistently associated with violent events in the adult population. However, the causality between SSRI use and violent behaviour was never found. Several recent studies draw the attention to this hypothesis while they were inspired by several mass murderers in the United States.

**Objectives:**

A literature research on studies exploring the association between SSRI use and violent behaviour.

**Methods:**

The authors performed a literature search (1966–2020) using PubMed and Embase to review studies where a possible link between SSRI’s and violent behaviour in adults was assessed.

**Results:**

94 studies were identified, of which 6 studies were included. There is no association between the use of SSRI’s and violent behaviour in the general population. However, an increased hazard of violent behaviour was observed in young man and those with a history of violent crime.

**Conclusions:**

Overall, SSRI treatment is safe in the general population. Certain subgroups can, however, be vulnerable to aggressive flare-ups, especially during on-treatment period and the first 12 weeks after drug discontinuation. Therefore, careful monitoring throughout these critical periods is strongly recommended.

**Disclosure:**

No significant relationships.

